# Prevalence of Nontuberculous Mycobacteria among Extrapulmonary Tuberculosis Cases in Tertiary Care Centers in Northern India

**DOI:** 10.1155/2015/465403

**Published:** 2015-03-26

**Authors:** A. K. Maurya, V. L. Nag, S. Kant, R. A. S. Kushwaha, M. Kumar, A. K. Singh, T. N. Dhole

**Affiliations:** ^1^Department of Microbiology, All India Institute of Medical Sciences, Jodhpur, Rajasthan 342005, India; ^2^Department of Pulmonary Medicine, King George Medical University, Lucknow, Uttar Pradesh 226003, India; ^3^Department of Microbiology, Sanjay Gandhi Postgraduate Institute of Medical Sciences, Lucknow, Uttar Pradesh 226014, India

## Abstract

The reports of nontuberculous mycobacteria (NTM) associated with extrapulmonary diseases are increasing in tertiary care hospitals. Despite a significant increase in knowledge about NTM infections, they still represent a diagnostic and therapeutic challenge. The aim of this study is to know the prevalence of NTN among extrapulmonary tuberculosis cases in tertiary care centers in Northern India. A total of 227 culture positive isolates from 756 cases were tested for niacin production and catalase assay. BIO-LINE SD Ag MPT64 TB test and final identification and differentiation between MTBC and different species of NTM were further confirmed by GenoType Mycobacterium CM/AS assay. 71 cases (9.3%) were positive for AFB by ZN staining and 227 cases (30.1%) were positive for mycobacteria by culture. Niacin production and catalase activity were negative in 62/227 (27.4%) strains and after using a panel of different biochemicals and final confirmation by GenoType Mycobacterium CM assay. Out of 227 cultures tested, 165 (72.6%) strains were confirmed as *M. tuberculosis* complex, and 62 (27.4%) were confirmed as NTM. The most common NTM species identified were *M. fortuitum* 17 (27.5%) and *M. intracellulare* 13 (20.9%). The rapid identification of NTM species may help in targeted therapy and management of the diseases.

## 1. Introduction

Nontuberculous mycobacterium (NTM) has been observed for 100 years, but the trend of increasing prevalence of NTM is great concern for clinician as well as microbiologist. NTM are an important cause of morbidity and mortality in the progressive lung diseases [1]. More than 125 species of NTM have been catalogued and available out of which at least 42 species related with diseases in NTM [2]. American Thoracic Society and Infectious Diseases Society of America have developed guidelines for NTM disease determination, including clinical symptom, radiographic finding, and microbiologic criteria [3]. Clinical presentation of* Mycobacterium tuberculosis* complex (MTBC) and NTM may or may not be the same, but the treatment regimen is always different for both the infections [4]. Several species of NTM caused pulmonary and extrapulmonary diseases such as skin/soft-tissue infections and lymphatic, disseminated, and nosocomial infections after surgery of varying severity in both sporadic and epidemic form [5, 6]. Disseminated disease due to NTM is mostly related with AIDS and other forms of severe immunosuppression [3]. Conventional biochemical methods are able to identify mycobacterial species; but they are tedious and time consuming and require elaborate safety precautions. World Health Organization (WHO) recommended use of line probe assay to reduce the time for culture, identification, and drug resistance detection to as short as 2 days [7]. DNA strip technology (line probe assays) based on the reverse hybridization of PCR products to their complementary probes has been used for the simultaneous detection and identification of mycobacteria. The GenoType Mycobacterium common mycobacteria/additional species (CM/AS) assay (Hain Lifescience, Nehren, Germany) is a new commercial kit developed to differentiate and identify different species of NTM from cultures. It involves DNA amplification targeting the 23S rRNA gene region, followed by reverse hybridization to specific oligonucleotide probes immobilized on membrane strips. There are two kits; the CM identifies 15* Mycobacterium* species, including* M. tuberculosis* complex, while the AS kit aims to differentiate 16 additional less common NTM species available for differentiation and this assay is very accurate and reliable test for the identification of mycobacterial species [8, 9]. The prevalence of NTM and identification rate in pulmonary diseases varied from 0.7% to 34% in India [9–11]. The increases in frequency of NTM infection make this emerging infectious disease relevant to all pulmonary/extrapulmonary diseases and clinical practitioners in India as well as abroad. There is a lack of recent and widespread data regarding the frequency of NTM infection in extrapulmonary tuberculosis (EPTB) cases in tertiary care hospitals in India. The present study has been conducted to know prevalence of NTM among extrapulmonary tuberculosis cases in tertiary care centers in Northern region of India.

## 2. Methods

### 2.1. Study Design

The study was performed prospectively.

### 2.2. Clinical Specimens and Data Collection

Specimens were collected from Kasturba Chest Hospital, Department of Pulmonary Medicine, King George Medical University, Uttar Pradesh, Lucknow, India, and from various outpatient department and wards of SGPGIMS, Lucknow, India. 2–10 mL of nonrepeated specimens were collected from 756 suspected cases of EPTB. The specimens were included as lymph node aspirate and cold abscesses, pleural fluid, cerebrospinal fluid, synovial fluid, ascitic fluid, urine, gastric aspirate, pus, bone marrow aspirates, wound swabs, and biopsy materials. All the patients signed due consent for sample collection. The clinical history regarding present and past history of antitubercular treatment (ATT) and family history of tuberculosis and any other associated disease were taken in prescribed Performa.

### 2.3. Processing and Microbiological Analysis of Specimens

All the clinical specimens were grouped into two types based on presence and absence of normal commensal bacterial flora as nonsterile and sterile specimens. Nonsterile specimens (respiratory) were processed as per standard N-acetyl-L-cysteine-NaOH method [12] and sterile specimens were processed as per standard laboratory protocol (quantity >0.5 mL was centrifuged and <0.5 mL was directly inoculated) in Class II Biosafety cabinet. After specimens processing, centrifuged pellets were subjected to smear microscopy by Ziehl-Neelsen (ZN) staining method and 0.5 mL was inoculated into BacT/ALERT MP vials in the BacT/ALERT 3D system (bioMerieux, France) containing modified Middlebrook 7H9 with an antibiotic supplement [13]. The reading of BacT/ALERT MP vials was monitored continuously by the BacT/ALERT 3D system [14]. Positive vials were subjected to smear microscopy for the presence of acid fast bacilli (AFB). No growth after 6 weeks of incubation was treated as negative for mycobacteria. Growth of different* Mycobacterium* species was differentiated based on a battery of tests which includes niacin production, catalase activity at 68°C at pH 7, and p-nitrobenzoic acid (PNB) [15]. BBL Taxo TB Niacin Test Strips (Becton and Dickinson, USA), absorbent paper strips, and TB Niacin positive test control paper discs were used according to the manufacturer's instruction. The catalase assay for mycobacteria was performed using 30% H_2_O_2_ (Superoxol) in 10% Tween-80 and the test performed at both 22–25°C and 68°C [16].

### 2.4. BIO-LINE SD Ag MPT64 TB Test

A total volume of 100 *μ*L of broth from BacT/ALERT culture was added to the well of BIO-LINE SD Ag MPT64 TB rapid kit cassette. Inoculated immunochromatographic cassettes were kept at room temperature for 20 min in Class II Biosafety cabinet and examined for the presence of control and test band. Appearance of the band in the “C” region confirmed the validity of the test. Additional appearance of the band in the “T” region was interpreted as positive for the presence of MPT64 Ag antigen in the test broth culture. No band in the “C” region was interpreted as invalid test [4, 8]. Standard reference strain* M. tuberculosis* complex H37Rv ATCC 27294 was used as a positive control.

### 2.5. GenoType Mycobacterium CM/AS Assay

All isolates were identified using conventional biochemical techniques [9] before molecular identification by the GenoType Mycobacterium CM/AS assay. The final confirmation of MTBC and NTM species was done by the GenoType Mycobacterium CM/AS assay of BacT/ALERT MP culture positive mycobacterial isolates [9, 17]. The presence of distinct band at positions 10 and 16 indicated a positive test for* M. tuberculosis* complex. Standard strain of* M. tuberculosis* complex H37* Rv* ATCC 27294,* Mycobacterium fortuitum* ATCC 6841,* Mycobacterium intracellulare* ATCC 13950, and* M. abscessus* ATCC 19977 was used as control in this study.

### 2.6. Ethics Statement

The study was approved by the Institutional Ethical Committee of King George Medical University Uttar Pradesh, Lucknow, India (Approval no.: XXVIII extracellular matrix-B/P2). A written informed consent was obtained from all subjects before enrollment into the study.

## 3. Results

### 3.1. Smear Microscopy and Culture of Mycobacteria

Out of 756 EPTB specimens, 71 (9.3%) were positive for AFB by ZN staining and 227 (30.1%) were positive for mycobacteria by culture. 165/227 (20.6%) were confirmed as* M. tuberculosis* complex by the biochemical and molecular test. The history of contact with TB patients was determined in 37 cases (22.4%), history of diabetes mellitus was found in 19 (11.5%), family history of TB was present in 23 (13.9%), and 3 (1.8%) cases were HIV positive.

### 3.2. Identification and Confirmation of NTM by Conventional and Molecular Methods

Phenotypically identification of NTM by niacin production and catalase assay were negative in 62/227 (27.4%). After using a panel of different biochemicals and again testing by rapid BIO-LINE SD Ag MPT64 TB test for rapid differentiation between MTBC and NTM and using confirmatory (gold standard) test by GenoType Mycobacterium CM/AS assay, 165 (72.6%) isolates were* M. tuberculosis* complex and the remaining 62 (27.4%) isolates were identified as different species of NTM by GenoType Mycobacterium CM/AS assay.

### 3.3. Prevalence of NTM Infection by GenoType Mycobacterium CM/AS Assay

In this study, 62 (27.4%) NTM isolates available for analysis yielded 62 identifiable NTM by the GenoType CM/AS assay. We found that the most common NTM species identified were* M. fortuitum* 17 (27.5%),* M. intracellulare* 13 (20.9%),* M. abscessus* 9 (14.6%),* M. chelonae* 8 (12.9%),* M. avium* complex 5 (8.1%), and* M. kansasii* 3 (4.8%) as shown in Table 1.

## 4. Discussion

NTM have been increasingly recognized as an important cause of morbidity in the developing countries. The identification of NTM is important because positive microscopy cannot differentiate* M. tuberculosis* complex from NTM infection, causing diagnostic and clinical dilemmas. Management of patients with MTBC and NTM is entirely different; therefore prompt isolation, detection, and differentiation are necessary for suitable management [9, 18]. The increases in NTM isolated, sensitization, and disease prevalence have been noted worldwide as well as in India [19–26]. The species of NTM isolated from humans are also present in soil and natural open water sources, all of which play a key role as sources of human infections [27]. In present study the rapidly growing mycobacteria were prominently isolated from extrapulmonary samples;* M. fortuitum* (27.4%) and* M. abscessus* (14.4%) were the most frequently isolated species. Various studies revealed that isolation frequency of NTM species may differ according to the geographical region in which species were obtained as well as clinical material, clinical manifestation, and underlying disease of the patient [28–32]. American Thoracic Society (ATS) guidelines recommended course of 12–18 months for slow-growing mycobacteria, such as* M. kansasii* or* M. avium* complex (MAC), and longer courses for rapidly growing mycobacteria [32].

In the present study* M. abscessus* (14.4%) was the second most frequently isolated species from extrapulmonary samples. The treatment of NTM infections associated with respiratory infection caused by* M. abscessus* is difficult in comparison to other species [30, 33–35]. Various studies from India showed NTM prevalence of 17.4% from clinical specimens in patients with fibrocavitary disease and 7.4% from various clinical specimens;* M. fortuitum* was the commonest isolate, 8.6% of NTM from sputum specimens in patients with BCG trial area and reported 8.3%–34% of NTM infection in different studies from Delhi and Kasauli, [10, 11, 25, 26, 36]. In contrast to previous few studies,* M. fortuitum* 17 (27.5%) was most common followed by* M. intracellulare* 13 (20.9%),* M. abscessus* 9 (14.6%),* M. chelonae* 8 (12.9%),* M. avium* complex 5 (8.1%), and* M. kansasii* 3 (4.8%) were isolated in the present study.

Conventional biochemical tests used to identify different mycobacterial species are complex and time consuming [37]; various genetic probes and newer techniques like high-performance liquid chromatography, chemiluminescent deoxyribonucleic acid (DNA) probes, nucleic acid amplification, and sequencing of 16S ribosomal ribonucleic acid (rRNA) genes are more sophisticated and highly expensive and require expensive equipment [8, 38]. The GenoType Mycobacterium common mycobacteria/additional species (CM/AS) assay is a new commercial kit developed to differentiate and identify different species of NTM from cultures. Our results showed that NTM isolates accounted for 27.4% of all isolates of mycobacteria identified in extrapulmonary specimens. Rapid speciation that distinguishes MTBC from NTM is an important prerequisite for the proper management of patients with mycobacterial infections and may be introduced as a required standard into routine laboratory diagnostics in tertiary care centers in developing counties.

## 5. Conclusion

The high prevalence (27.4%) of NTM which includes* M. fortuitum* 17 (27.5%) and* M. intracellulare* 13 (20.9%) was the most frequently isolated rapid growing mycobacteria in extrapulmonary tuberculosis cases in tertiary care centers in Northern India. Overall rapid identification and differentiation to species level by molecular assay may help in targeted therapy and management of infections caused by different mycobacterial species and indirectly, it will also help in reducing the developing of antimicrobial drug resistance among NTM isolates in the community.

## Figures and Tables

**Table 1:** Prevalence of NTM species differentiated by GenoType Mycobacterium CM/AS assay (*n* = 62).

Species of nontubercular mycobacteria	Frequency (%)
*M. fortuitum *	17 (27.5%)
*M. intracellulare *	13 (20.9%)
*M. abscessus *	9 (14.6%)
*M. chelonae *	8 (12.9%)
*M. avium* complex	5 (8.1%)
*M. kansasii *	3 (4.8%)
*M. interjectum *	2 (3.2%)
*M. gordonae *	2 (3.2%)
Other NTM	3 (4.8%)

NTM: nontuberculous mycobacteria, CM/AS: common mycobacteria/additional species, *M.  fortuitum*: *Mycobacterium  fortuitum*, *M.  intracellulare*: *Mycobacterium  intracellulare, M.  abscessus*: *Mycobacterium  abscessus*, *M.  chelonae*: *Mycobacterium  chelonae*, *M.  avium*: *Mycobacterium  avium*, *M.  kansasii*: *Mycobacterium  kansasii*, *M.  gordonae*: *Mycobacterium  gordonae*.

## References

[1] Field S. K., Fisher D., Cowie R. L. (2004). Mycobacterium avium complex pulmonary disease in patient without HIV infection. *Chest*.

[2] Tortoli E. (2003). Impact of genotypic studies on mycobacterial taxonomy: the new mycobacteria of the 1990s. *Clinical Microbiology Reviews*.

[3] Cassidy P. M., Hedberg K., Saulson A., McNelly E., Winthrop K. L. (2009). Nontuberculous mycobacterial disease prevalence and risk factors: a changing epidemiology. *Clinical Infectious Diseases*.

[4] Maurya A. K., Nag V. L., Kant S. (2012). Evaluation of an immunochromatographic test for discrimination between *Mycobacterium tuberculosis* complex & non tuberculous *mycobacteria* in clinical isolates from extra-pulmonary tuberculosis. *Indian Journal of Medical Research*.

[5] Horsburgh C. R. (1996). Epidemiology of disease caused by nontuberculous mycobacteria. *Seminars in Respiratory Infections*.

[6] Winthrop K. L., Abrams M., Yakrus M. (2002). An outbreak of mycobacterial furunculosis associated with footbaths at a nail salon. *The New England Journal of Medicine*.

[7] World Health Orgnization (2007). *The Use of Liquid Medium for Culture and Drug Susceptibility Testing (DST) in Low and Medium Income Settings: Summary of the Expert Group Meeting on the Use of liquid Culture Systems*.

[8] Lee A. S., Jelfs P., Sintchenko V., Gilbert G. L. (2009). Identification of non-tuberculous mycobacteria: utility of the GenoType Mycobacterium CM/AS assay compared with HPLC and 16S rRNA gene sequencing. *Journal of Medical Microbiology*.

[9] Singh A. K., Maurya A. K., Umrao J. (2013). Role of genotype((R)) mycobacterium common mycobacteria/additional species assay for rapid differentiation between *Mycobacterium tuberculosis* complex and different species of non-tuberculous mycobacteria. *Journal of Laboratory Physicians*.

[10] Paramasivan C. N., Govindan D., Prabhakar R., Somasundaram P. R., Subbammal S., Tripathy S. P. (1985). Species level identification of non-tuberculous mycobacteria from South Indian BCG trial area during 1981. *Tubercle*.

[11] Myneedu V. P., Verma A. K., Bhalla M. (2013). Occurrence of non-tuberculous mycobacterium in clinical samples—a potential pathogen. *Indian Journal of Tuberculosis*.

[12] Scott C. P., Filho L. D. F., Mello F. C. D. Q. (2002). Comparison of C_18_-carboxypropylbetaine and standard *N*-acetyl-L-cysteine-NaOH processing of respiratory specimens for increasing tuberculosis smear sensitivity in Brazil. *Journal of Clinical Microbiology*.

[13] Carricajo A., Fonsale N., Vautrin A. C., Aubert G. (2001). Evaluation of BacT/Alert 3D liquid culture system for recovery of mycobacteria from clinical specimens using sodium dodecyl (Lauryl) sulfate-NaOH decontamination. *Journal of Clinical Microbiology*.

[14] Ängeby K. A. K., Werngren J., Toro J. C., Hedström G., Petrini B., Hoffner S. E. (2003). Evaluation of the BacT/ALERT 3D system for recovery and drug susceptibility testing of *Mycobacterium tuberculosis*. *Clinical Microbiology and Infection*.

[15] Kubica G. P. (1973). Differential identification of mycobacteria. VII. Key features for identification of clinically significant mycobacteria. *American Review of Respiratory Disease*.

[16] Sharma B., Pal N., Malhotra B., Vyas L. (2010). Evaluation of a rapid differentiation test for *Mycobacterium tuberculosis* from other mycobacteria by selective inhibition with p-nitrobenzoic acid using MGIT 960. *Journal of Laboratory Physicians*.

[17] Richter E., Rüsch-Gerdes S., Hillemann D. (2006). Evaluation of the GenoType *Mycobacterium* assay for identification of mycobacterial species from cultures. *Journal of Clinical Microbiology*.

[18] Hasegawa N., Miura T., Ishii K. (2002). New simple and rapid test for culture confirmation of *Mycobacterium tuberculosis* complex: a multicenter study. *Journal of Clinical Microbiology*.

[19] Prevots D. R., Shaw P. A., Strickland D. (2010). Nontuberculous mycobacterial lung disease prevalence at four integrated health care delivery systems. *The American Journal of Respiratory and Critical Care Medicine*.

[20] Thomson R. M. (2010). Changing epidemiology of pulmonary nontuberculous mycobacteria infections. *Emerging Infectious Diseases*.

[21] O'Brien D. P., Currie B. J., Krause V. L. (2000). Nontuberculous mycobacterial disease in northern Australia: a case series and review of the literature. *Clinical Infectious Diseases*.

[22] Park Y. S., Lee C.-H., Lee S.-M. (2010). Rapid increase of non-tuberculous mycobacterial lung diseases at a tertiary referral hospital in South Korea. *International Journal of Tuberculosis and Lung Disease*.

[23] Marras T. K., Chedore P., Ying A. M., Jamieson F. (2007). Isolation prevalence of pulmonary non-tuberculous mycobacteria in Ontario, 1997–2003. *Thorax*.

[24] Jesudason M. V., Gladstone P. (2005). Non tuberculous mycobacteria isolated from clinical specimens at a tertiary care hospital in South India. *Indian Journal of Medical Microbiology*.

[25] Chakrabarti A., Sharma M., Dubey M. L. (1990). Isolation rates of different mycobacterial species from Chandigarh (North India). *Indian Journal of Medical Research—Section A Infectious Diseases*.

[26] Karak K., Bhattacharyya S., Majumdar S., de P. K. (1996). Pulmonary infection caused by Mycobacteria other than *M. tuberculosis* in and around Calcutta. *Indian Journal of Pathology and Microbiology*.

[27] van Ingen J., Boeree M. J., Dekhuijzen P. N. R., van Soolingen D. (2009). Environmental sources of rapid growing nontuberculous mycobacteria causing disease in humans. *Clinical Microbiology and Infection*.

[28] Gunaydin M., Yanik K., Eroglu C. (2013). Distribution of nontuberculous Mycobacteria strains. *Annals of Clinical Microbiology and Antimicrobials*.

[29] de Groote M. A., Huitt G. (2006). Infections due to rapidly growing mycobacteria. *Clinical Infectious Diseases*.

[30] Falkinham J. O. (1996). Epidemiology of infection by nontuberculous mycobacteria. *Clinical Microbiology Reviews*.

[31] Yang S.-C., Hsueh P.-R., Lai H.-C. (2003). High prevalence of antimicrobial resistance in rapidly growing mycobacteria in Taiwan. *Antimicrobial Agents and Chemotherapy*.

[32] de Mello K. G. C., Queiroz Mello F. C., Borga L. (2013). Clinical and therapeutic features of pulmonary nontuberculous mycobacterial disease, Brazil, 1993—2011. *Emerging Infectious Diseases*.

[33] Jarzembowski J. A., Young M. B. (2008). Nontuberculous mycobacterial infections. *Archives of Pathology and Laboratory Medicine*.

[34] Koneman E. W., Allen S. D., Janda W. M. (2006). *Color Atlas and Textbook of Diagnostic Microbiology*.

[35] Huang C.-T., Tsai Y.-J., Shu C.-C. (2009). Clinical significance of isolation of nontuberculous mycobacteria in pulmonary tuberculosis patients. *Respiratory Medicine*.

[36] Das B. K., Sharma V. K., Rao Bhau L. N., Saxena S. N., Bhardwaj B. K. (1982). Characterisation of mycobacterial strains from clinical specimens. *Indian Journal of Pathology and Microbiology*.

[37] Marzouk M., Kahla I. B., Hannachi N. (2011). Evaluation of an immunochromatographic assay for rapid identificationof *Mycobacterium tuberculosis* complex in clinical isolates. *Diagnostic Microbiology and Infectious Disease*.

[38] Ichiyama S., Iinuma Y., Yamori S., Hasegawa Y., Shimokata K., Nakashima N. (1997). Mycobacterium growth indicator tube testing in conjunction with the AccuProbe or the AMPLICOR-PCR assay for detecting and identifying mycobacteria from sputum samples. *Journal of Clinical Microbiology*.

